# SRC and TP53 play critical role in low-grade dysplasia colorectal mucosa transformation into cancer 

**Published:** 2018

**Authors:** Hamid Asadzadeh-Aghdaei, Mona Zamanian Azodi, Reza Vafaee, Hamideh Moravvej Farshi, Nosratollah Naderi

**Affiliations:** 1 *Basic and Molecular Epidemiology of Gastrointestinal Disorders Research Center, Research Institute for Gastroenterology and Liver Diseases, Shahid Beheshti University of Medical Sciences, Tehran, Iran.*; 2 *Student Research Committee, Proteomics Research Center, Faculty of Paramedical Sciences, Shahid Beheshti University of Medical Sciences, Tehran, Iran*; 3 *Proteomics Research Center, Shahid Beheshti University of Medical Sciences, Tehran, Iran*; 4 *Skin Research Center, Shahid Beheshti University of Medical Sciences, Tehran, Iran*; 5 *Gastroenterology and Liver Diseases Research Center, Research Institute for Gastroenterology and Liver Diseases, Shahid Beheshti University of Medical Sciences, Tehran, Iran*

**Keywords:** Dysplasia lesion, Colorectal cancer, Protein-protein interaction network analysis

## Abstract

**Aim:**

Determination of crucial genes that are involved in onset and progress of dysplasia of colorectal mucosa is the aim of this study.

**Background:**

Management of dysplasia as one of the risk factors of colon cancer is very challenging. Molecular studies could be helpful in this matter. Here, the transcriptome profile of low-grade dysplasia in colon tissue in comparison with normal one is studied by protein-protein interaction (PPI) network analysis.

**Methods:**

For detecting differentially expressed genes (DEGs) of dysplasia lesion, the data was downloaded from the gene chip GSE31106, platform GPL1261, GSM770092-94 as normal colorectal mucosa group and GSM770098-100 as low-grade dysplasia colorectal mucosa from the Gene Expression Omnibus database (GEO). The expression profile is evaluated by GEO2R and a network of DEGs is constructed and analyzed by Cytoscape algorithms.

**Results:**

The findings indicate that a PPI network analysis of 113 DEGs is consist of 8 nodes that 6 of them are common with inflammation state. Only SRC and TP53 were recognized as the specific makers for dysplasia. In this respect, a subnetwork of these two genes introduce a panel of 8 nodes consist of HRAS, MYC, PIK3CA, PIK3CB, PIK3CD, PIK3CG, SRC, and TP53.

**Conclusion:**

It can be concluded that SRC and TP53 may play prominent role in dysplasia pathogenicity after running validation tests.

## Introduction

 Cancer of colon is the most frequent cause of cancer death worldly (1). Long-standing inflammatory bowel disease (IBD) is a risk factor for colon cancer as two different types including ulcerative colitis (UC) and Crohn’s disease (2). In addition, prior to tumor formation, dysplasia lesions could develop from IBD with different grades known as low and high grades (3). In view of this fact, the cancer management requires precancerous dysplasia identification via surveillance approaches known as colonoscopy (4). However, it is known that dysplasia detection is complicated especially for the low-grade type (5). The reason for this is due to hardship of differentiate from normal tissue and their multifocal properties (6). A requirement for establishing methods of accurate and less invasive approaches for the diagnosis of low-grade dysplasia for the treatment approaches, attracted researchers to the molecular studies. This can be achievable through high-throughput investigations. In this respect, sequential analysis of tumorigenesis from inflammation to the tumor could assist improving the understanding the underlying mechanisms from molecular perspective (7). One way to assist this, is application of bioinformatic study such as interaction analysis. Interaction mapping could promote introducing the most prominent biomarkers of the disease (8). In fact, aiming toward a better understanding of dysplasia and ultimately tumorigenesis from molecular view (7), suggested protein-protein interaction (PPI) network analysis by this research. In this approach, the involved genes in disorder were screened and the prominent ones introduced as possible biomarkers (9, 10). In other words, biomarker discovery in dysplasia could be helpful for the prediction of colon cancer. Here, the interactome profile of dysplasia lesion is compared and evaluated with normal condition. 

## Methods

**Data collection**

Series GSE31106, platform GPL1261, GSM770092-94 as normal colorectal mucosa group and GSM770098-100 as low-grade dysplasia colorectal mucosa group were downloaded from GEO database. 10mg/kg Azoxymethane was intraperitoneal injected to the five-week-old male mice. The mice were treated with three cycles of Dextran sulfate sodium (2%, 1.5%, and 1.5%). Saline injection and distilled water drinking were applied for normal control group. Microscopic assessment of colorectal tissue confirmed low-grade dysplasia in treated samples related to the normal group. Affymerix GeneChip Mouse Genome 430 2.0 Array was used to evaluate extracted RNAs of tissue. Based on p-value ≤ 0.05 and 2 ≤ fold change ≤ 0.5 the 250 top DEGs were selected to further analysis.

**PPI network analysis**

The characterized and significant DEGs were interacted by STRING (11) database plugin of Cytoscape software v 6.3.2 (12). The constructed network was analyzed by Network Analyzer application of Cytoscape and hubs are determined as the nodes with degree value ≥ (mean+2SD) (13). Since similar research is dealing about mice that are treated like these mice but investigated two weeks later and were diagnosed as inflamed colorectal samples, the hubs and 50 relative genes which probably differentiate dysplasia network from inflamed one were included in a PPI sub-network. The constructed sub-network was analyzed to introduce possible mechanism of transition from inflamed colorectal to low grade dysplasia tissue. 

**Statistical analysis**

Gene expression profiles were matched via boxplot analysis and p-value <0.05 was considered for significant findings. 

## Results

**Figure 1:**
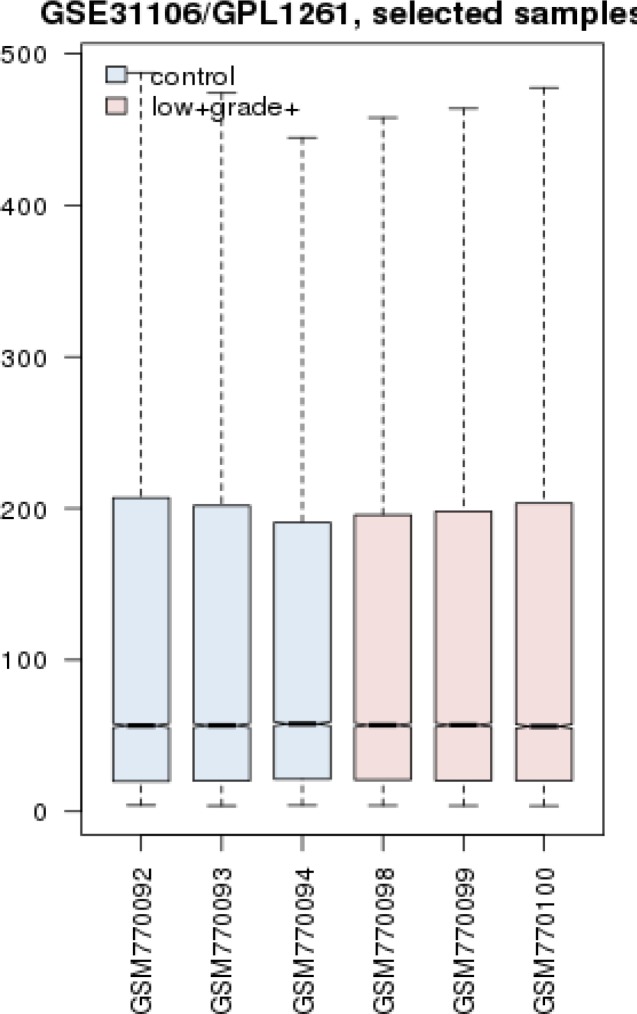
Boxplot analysis shows that the gene expression profiles of low-grade dysplasia samples and controls are matched

Gene profiles of the normal controls and low-grade dysplasia were matched via boxplot analysis to promote more exploration (Figure 1). 

**Figure 2:**
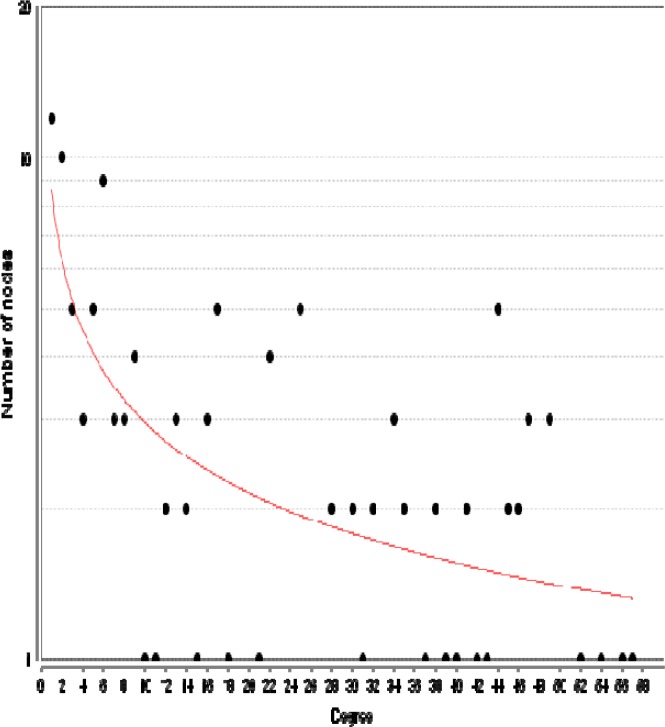
Degree distribution for nodes of PPI network

Among 250 top significant DEGs, 158 individuals were characterized which were included in the constructed PPI network. A number of 113 DEGs were recognized by STRING database and 45 ones were not documented. The network was created by 113 DEGs and 50 related genes. The network included 40 isolated nodes, one paired genes and a main connected component which was characterized with 121 nodes and 1190 edges. The network was analyzed and it was confirmed that the built network is scale free. Degree distribution of network (Figure 2) was fitted to equation y = ax^b^ where a and b were 8.615 and -0.464, respectively. 

**Figure 3:**
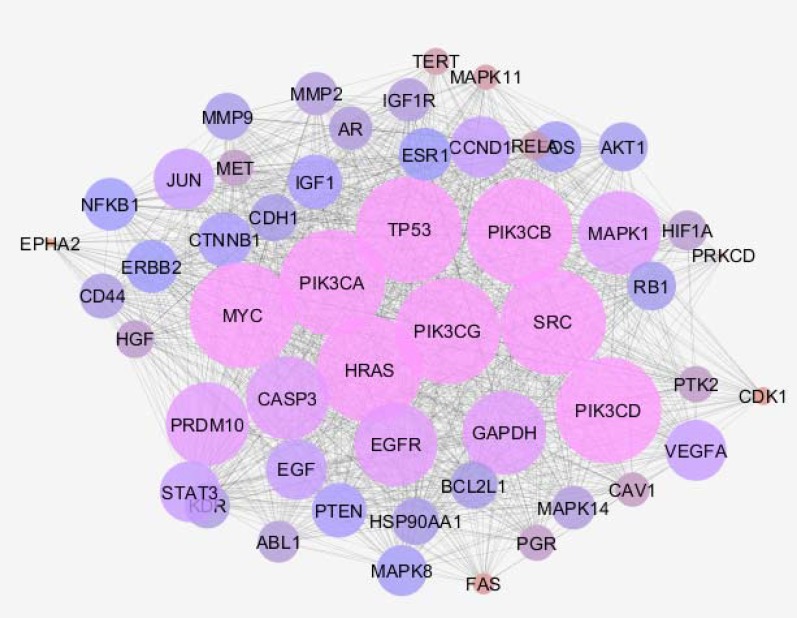
PPI sub-network of SRC, TP53, and 50 related genes. The nodes are layouted based on degree value. The bigger the size the higher the degree value; likewise, color changes from blue to red refer to higher degree values

This equation refers to scale free network (14). Correlation between data and R-squired (it was computed on logarithmaized values) were 0.789 and 0.404, respectively. As it is shown in the table 1, 8 hub nodes of network including PRDM10, GAPDH, INS, AKT1, SRC, IL6, ALB, and TP53 were determined. Data from PPI network analysis of inflamed samples (the mice which were treated as like the samples of this research but investigated two weeks after treatment) revealed PRDM10, GAPDH, INS, AKT1, IL6, and ALB are hub nodes. Hence, SRC and TP53 differentiate the two researches. Sub-network of SRC, TP53, and 50 related genes and the hubs of the sub-network are shown in the figure 3 and table 2, respectively.

**Table 1:** Hub nodes from the DEGs PPI network are tabulated

R	display name	description	Degree
1	PRDM10	PR domain containing 10	57
2	GAPDH	glyceraldehyde-3-phosphate dehydrogenase	56
3	INS	insulin	54
4	AKT1	v-akt murine thymoma viral oncogene homolog 1	52
5	SRC	v-src sarcoma (Schmidt-Ruppin A-2) viral oncogene homolog (avian)	49
6	IL6	interleukin 6 (interferon, beta 2)	49
7	ALB	albumin	49
8	TP53	tumor protein p53	47

**Table 2:** Hub nodes of PPI sub-network of SRC, TP53, and 50 related genes

R	Gene name	Description	Degree
1	HRAS	v-Ha-ras Harvey rat sarcoma viral oncogene homolog	51
2	MYC	v-myc myelocytomatosis viral oncogene homolog (avian)	51
3-1	PIK3CA	phosphatidylinositol-4,5-bisphosphate 3-kinase, catalytic subunit alpha	51
3-2	PIK3CB	phosphatidylinositol-4,5-bisphosphate 3-kinase, catalytic subunit beta	51
3-3	PIK3CD	phosphatidylinositol-4,5-bisphosphate 3-kinase, catalytic subunit delta	51
3-4	PIK3CG	phosphatidylinositol-4,5-bisphosphate 3-kinase, catalytic subunit gamma	51
4	SRC	v-src sarcoma (Schmidt-Ruppin A-2) viral oncogene homolog (avian)	51
5	TP53	tumor protein p53	51

## Discussion

Molecular studies showed promising for understanding disease behavior and mechanisms of which, Introduction of biomarkers could be helpful for diagnosis, screening, and treatment protocols. One way is the study of genome profile of disease state in comparison with the normal condition to recognize the differentially expressed genes. These genes could also be more promising in terms of biomarker study, if further evaluated for their interaction properties in a whole interaction system known as PPI map (15). Here, we followed this procedure and genes that were modified in expression in dysplasia lesion were analyzed and used for interactomic investigation. What is more, low-grade dysplasia lesions are important to be examined for their valuable asset of being potential for developing cancerous condition (16). It is durable to detect low-grade dysplasia, in this respect, molecular biomarker discovery can provide novel information and facilitate both the detection and intervention approaches. For perusing this goal, in this conducted research, we first analyzed and compared the groups of samples in terms of expression values and it was concluded that these groups are comparable statistically as demonstrated in figure 1. The next step was the determination of DEGs and the construction a network of protein interaction. The DEGS shaped a network in which, 8 nodes declared central values known as hub-bottleneck elements. These 8 nodes includes PRDM10, GAPDH, INS, AKT1, SRC, IL6, ALB, and TP53 that among them 6 ones are common with inflammation based on the previous study conducted by the same research group. SRC and TP53 are the uncommon ones that apparently specific to dysplasia. It seems that SRC and TP53 act in the opposite manner including tumorigenesis and tumor suppressor activity respectively. Therefore, elevation of SRC and decrement of TP53 are expected. Expression change of these two important genes in low-grade dysplasia can be interpreted as potential of dysplasia to convert into colon cancer. To gain a better view of possible involved biomarkers of dysplasia, a sub-network of the 2 probable agents was constructed and some other genes are explored. The second set of hub-bottlenecks derived from SRC and TP53 sub-network are as follow, HRAS, MYC, PIK3CA, PIK3CB, PIK3CD, PIK3CG, SRC, and TP53. As it is clear from table 2, all the genes possesses similar degree values. First, there is a family of gene named PIK3C (A, B, C, D, and G). Five members of this gene set are available in this sub-network. There are some evidence that PIK3CA mutations associated with activation of KRAS- BRAF mutation occurring in colorectal cancers (17). The other ones, HRAS, MYC, and the two last ones are our query genes (SRC and TP53). Allgayr *et al. *reported the prominent role of Src kinase activation in primary colorectal carcinoma (18). TP53 is a well-known gene which its deregulation is associated with many types of cancers especially colorectal cancers (19). TP53 was introduced as a common biomarker between colon and breast cancers (9). Chen *et al.* studied signal pathways related to SRC, TP53, and PIK3CA in colon cancer and deregulation of the genes were highlighted (20). Taking together this information can implies that the most of these genes are the famous parts of cancer pathogenicity. HRAS, MYC, and TP53 are known for their exceptional role in cancer growth and are studied highly in this field. Thus, these common biomarkers of cancer condition are also present in our sub-network of dysplasia. This may show that the dysplasia is in high risk for cancer trigger. A deeper look to the suggested panel may imply that quantities analysis of PIK3C, SRC, and TP53 expression be required as useful tool to follow up of dysplasia patients to prevent progress of lesion into cancer (21-24). 

In summary, PIK3C, SRC, and TP53 genes appeared as important common agents between low grade dysplasia and colon cancer. Due to participation of these genes in many different types of cancers, quantity analysis of gene expression of PIK3C, SRC, and TP53 in dysplasia colorectal condition can be beneficial.
